# Photosensitive lichenoid skin reaction to capecitabine

**DOI:** 10.1186/s12885-017-3882-4

**Published:** 2017-12-19

**Authors:** Rena A. Shah, Daniel D. Bennett, Mark E. Burkard

**Affiliations:** 1 Hematology/Oncology Department of Medicine, University of Wisconsin – Madison, Madison, WI USA; 2 Dermatology Department, University of Wisconsin – Madison, Madison, WI USA

**Keywords:** Capecitabine, Rash, Lichenoid rash, Photosensitive, Drug rash, Breast cancer

## Abstract

**Background:**

Capecitabine is an oral prodrug of fluorouracil, which is a common agent used in the management of many solid tumor malignancies. Dermatologic reaction is common with various chemotherapy agents but is not commonly reported in the use of capecitabine. When adverse reactions of rashes occur, the offending agent is typically removed. We report here an unusual case of photosensitive lichenoid rash due to capecitabine which is managed conservatively without major alteration in treatment.

**Case presentation:**

Seventy-three year old female with a diagnosis of stage IV breast cancer undergoing management with capecitabine presents with a rash during the summer months that is biopsy proven to be lichenoid photosensitive rash with likely offending agent being capecitabine. Her treatment was initially held despite having response to treatment, started on topical steroids after evaluation by dermatology. Given her response to treatment, drug was resumed with instructions to use sun precaution, sunscreen, and to complete course of topical steroids until rash resolution.

**Conclusion:**

Drug-related rashes tend to lead to disruptions or alterations in treatments of malignancies, despite responses. Given the wide use of capecitabine in many different solid tumors, it is important to recognize this photosensitive related skin rash and to initiate appropriate precautions of sun safety and topical steroids to allow minimal disruptions in therapy and continue use of capecitabine.

## Background

Photosensitivity is a common effect of certain aromatic chemotherapies such as doxorubicin. Here we report a case of atypical photosensitive lichenoid rash apparently caused by capecitabine. Photosensitive skin reactions are important to recognize because they can be managed by topical steroids and limiting exposure to ultraviolet light. It is important for clinicians to recognize this rare skin reaction to seek appropriate treatment without withholding potentially life-saving treatment.

Capecitabine is an oral prodrug of fluorouracil (5-FU), commonly used for solid tumor malignancies such as gastrointestinal cancers and laryngeal cancer. It is an effective single agent in metastatic breast cancer, and can be given in combination with docetaxel or lapatinib [1, 2]. Common adverse reactions to capecitabine include diarrhea and nausea, fatigue and elevated bilirubin, and hand-and-foot syndrome. Other rashes are rare [3].

## Case presentation

A 73 year old female with stage IV hormone-sensitive breast cancer to bone presented with rash on the face and arms. She presented twelve years prior with a large right breast mass. She was treated with modified radical mastectomy revealing multifocal invasive lobular carcinoma with 25 of 25 lymph nodes involved with immunohistochemistry staining revealing ER-positivity, PR-positivity, and HER2-negativity. Staging workup revealed bone metastases and she was treated with anastrozole and zolendronic acid. After eight years, progression of osseous metastatic disease was noted and anastrozole was switched to exemestane 25 mg daily. Eighteen months later, progression in the bones occurred and she was treated with investigational orteronel 400 mg twice daily. Two years later, progression in the bone was noted and she initiated fulvestrant 500 mg intramuscular every month. After one year, progression was again noted in the bone, along with increased CA27-19 tumor marker which reached 358 U/ml. Fulvestrant was discontinued and patient was started on capecitabine 2500 mg twice daily for 14 days on and 7 days off for 21-day cycle at the beginning of summer. The patient had minimal symptoms, remained active, and spent much of the summer months out of doors. After 3 cycles, she developed a mild rash on the face, arms, but the tumor marker fell to 256 U/ml. Therapy was continued and after two additional cycles the rash worsened as the tumor marker continued to decline and imaging showed sclerosis of bony metastases, suggestive of healing. The rash appeared in a photo-distributed pattern but the patient was uncertain if the rash was worsened by sun exposure.

The rash was initially described as scattered macules and papules with thin raised plaques that were mildly pruritic on her cheeks and forearms. After the rash became more severe, capecitabine was held and she was referred to dermatology. Other medications at the time were zolendronic acid 4 mg IV every 3 months, calcium 600 mg/vitamin D 200 IU twice daily, guaifenesin, ondansetron, and acetaminophen as needed.

**Fig. 1:**
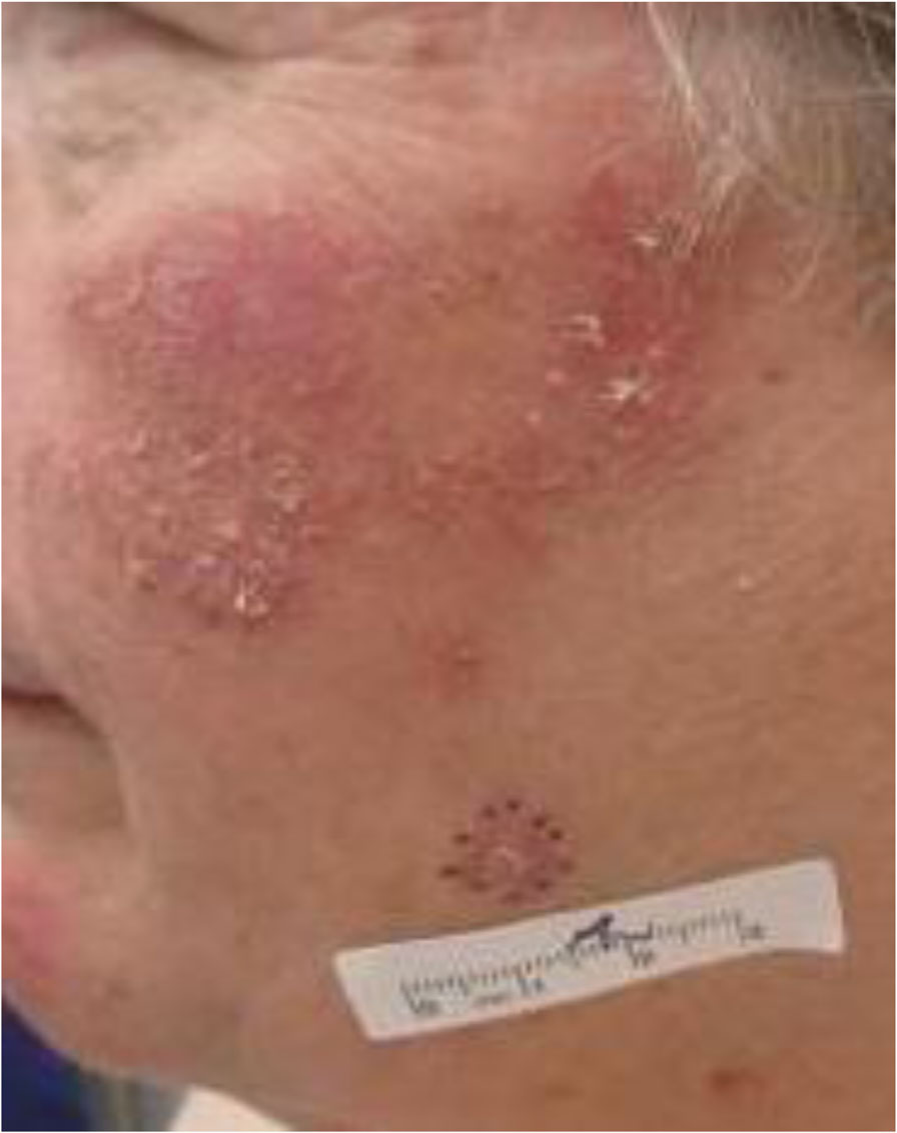
Clinical presentation of rash. Rash on left cheek

**Fig. 2:**
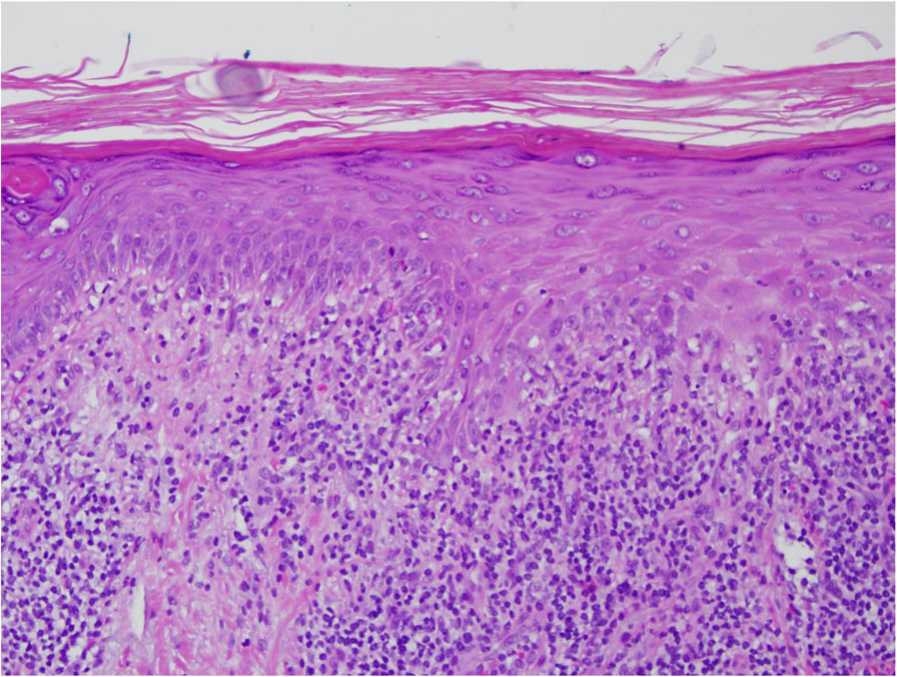
Histopathology of skin biopsy of rash (20X). Band-like inflammatory infiltrate with lichenoid features

Dermatologic evaluation revealed the rash remained localized to her face, upper chest, and forearms. (Fig. 1). Shave biopsies were obtained of lesions located on her left cheek, right shoulder, and left upper arm. All three biopsies demonstrated a lichenoid tissue reaction with rare eosinophils. The histopathologic differential diagnosis included lichen planus and lichenoid drug eruption, but the presence of eosinophils supported a diagnosis of a medication reaction. Clinically, this patient’s presentation is most consistent with photosensitive lichenoid drug eruption, most likely to capecitabine. (Fig. 2). She was treated with topical triamcinolone 0.1% ointment to arms and chest twice daily, hydrocortisone 2.5% ointment twice daily to her face with instructions to limit sun exposure, and to use regular sunscreen. She did not undergo any phototesting because it was not felt to be clinically indicated in this case. She was evaluated about 2 weeks after starting steroid treatment and was noted for improvement in her rash and capecitabine was resumed with sun protective measures and without recurrence of the rash.

## Discussion

Photosensitive lichenoid skin reaction is a rare skin manifestation of capecitabine and is reported twice in the dermatologic literature [4, 5]. In the reported cases, the rash appeared after the second or third cycle, usually in sun-exposed areas of the skin. Initially, the reaction manifests itself as a pruritic eruption, most commonly appearing in the summer months. In one case, capecitabine was discontinued and the rash resolved, but in the other, capecitabine was continued and the rash resolved with topical corticosteroids and sun protection. It is important to recognize that discontinuation of the drug was not necessary and rash was managed with topical treatments.

## Conclusion

Capecitabine-induced dermatologic reactions are most commonly associated with hand-and-foot syndrome but have also been implicated in oral lichenoid stomatitis [6], lupus erythematosus, and leukocytoclastic vasculitis [7], along with lichenoid skin eruption. Another oral fluorouracil agent, specifically tegafur, has also been implicated in development of lichenoid skin reaction [8]. Oral fluorouracil allows patients to continue chemotherapy without spending unnecessary time in infusion centers and hospitals, improving quality of life. With the increasing use of capecitabine, it is important to recognize various skin manifestations of the drug along with potential treatments that would preclude the need for an alternate therapy.

## Abbreviations

5-FU: Fluorouracil
ER: Estrogen receptor
HER2+: Her-2/neu receptor
PR: Progesterone receptor
